# Etiology of Pediatric Bacterial Meningitis Pre- and Post-PCV13 Introduction Among Children Under 5 Years Old in Lomé, Togo

**DOI:** 10.1093/cid/ciz473

**Published:** 2019-08-30

**Authors:** Enyonam Tsolenyanu, Rowan E Bancroft, Abdul K Sesay, Madikay Senghore, Mawouto Fiawoo, Djatougbe Akolly, Mawussi A Godonou, Novissi Tsogbale, Segla D Tigossou, Leopold Tientcheu, Anoumou Dagnra, Yawo Atakouma, Peter Sylvanus Ndow, Archibald Worwui, Dadja E Landoh, Jason M Mwenda, Joseph N Biey, Bernard Ntsama, Brenda A Kwambana-Adams, Martin Antonio

**Affiliations:** 1 Department of Paediatrics, Sylvanus Olympio Teaching Hospital, Lomé, Togo; 2 World Health Organization (WHO) Collaborating Centre for New Vaccines Surveillance, Medical Research Council Unit The Gambia at London School of Hygiene & Tropical Medicine, Banjul; 3 Department of Microbiology, Sylvanus Olympio Teaching Hospital, Lomé, Togo; 4 WHO Country Office of Togo, Lomé, Togo; 5 WHO Regional Office for Africa WHO/AFRO, Republic of Congo, Brazzaville; 6 WHO Intercountry Support Team West Africa, Ouagadougou, Burkina Faso; 7 Microbiology and Infection Unit, Warwick Medical School, University of Warwick, Coventry, United Kingdom

**Keywords:** Pneumococcus, meningitis, vaccine impact, pediatric, Togo

## Abstract

**Background:**

Pediatric bacterial meningitis (PBM) causes severe morbidity and mortality within Togo. Thus, as a member of the World Health Organization coordinated Invasive Bacterial Vaccine Preventable Diseases network, Togo conducts surveillance targeting *Streptococcus pneumoniae* (pneumococcus)*, Neisseria meningitidis* (meningococcus), and *Haemophilus influenzae*, at a sentinel hospital within the capital city, Lomé, in the southernmost Maritime region.

**Methods:**

Cerebrospinal fluid was collected from children <5 years with suspected PBM admitted to the Sylvanus Olympio Teaching Hospital. Phenotypic detection of pneumococcus, meningococcus, *and H. influenzae* was confirmed through microbiological techniques. Samples were shipped to the Regional Reference Laboratory to corroborate results by species-specific polymerase chain reaction.

**Results:**

Overall, 3644 suspected PBM cases were reported, and 98 cases (2.7%: 98/3644) were confirmed bacterial meningitis. Pneumococcus was responsible for most infections (67.3%: 66/98), followed by *H. influenzae* (23.5%: 23/98) and meningococcus (9.2%: 9/98). The number of pneumococcal meningitis cases decreased by 88.1% (52/59) postvaccine introduction with 59 cases from July 2010 to June 2014 and 7 cases from July 2014 to June 2016. However, 5 cases caused by nonvaccine serotypes were observed. Fewer PBM cases caused by vaccine serotypes were observed in infants <1 year compared to children 2–5 years.

**Conclusions:**

Routine surveillance showed that PCV13 vaccination is effective in preventing pneumococcal meningitis among children <5 years of age in the Maritime region. This complements the MenAfriVac vaccination against meningococcal serogroup A to prevent meningitis outbreaks in the northern region of Togo. Continued surveillance is vital for estimating the prevalence of PBM, determining vaccine impact, and anticipating epidemics in Togo.

## Teaser

*Streptococcus pneumoniae* was the predominant pathogen responsible for pediatric bacterial meningitis during longitudinal sentinel surveillance within Togo, from 2010 to 2016. However, pneumococcal meningitis cases decreased by 88.1% (52/59) postvaccine introduction; from 59 cases prevaccine introduction to 7 cases postvaccine introduction.

In 2014, the World Health Organization (WHO) reported that bacterial meningitis epidemics affect approximately 400 million people living within the geographical region of Africa known as the “meningitis belt” [1]. Togo is situated in West Africa, between Ghana and Benin, located in the heart of the meningitis belt. In 2016, the northern region of the country experienced a bacterial meningitis outbreak, with a reported 1975 cases and a consequent 127 deaths [2]. Moreover, between January and February 2017, more than 250 suspected cases of meningitis were reported in Togo, with 20 deaths and at least 14 cases confirmed to be caused by bacterial pathogens [2]. The 3 most common pathogens implicated in bacterial meningitis cases can be prevented by vaccination: *Streptococcus pneumoniae* (Pneumococcus), *Haemophilus influenzae* type b (Hib), and *Neisseria meningitidis* (meningococcus) [3–6].

Vaccines targeting pneumococcus, meningococcus, and Hib are effective at reducing both the morbidity and mortality associated with childhood bacterial meningitis [6, 7]. Consequently, various formulations of the pneumococcal conjugate vaccine (PCV), meningococcal conjugate vaccine (MenAfriVac), and Hib vaccine have been rolled out in the 26 countries contained within the extended meningitis belt with funding from Gavi The Vaccine Alliance and the Meningitis Vaccine Project (MVP) [8–11]. The implementation of these routine conjugate vaccine programs has been hugely successful in reducing the burden of invasive bacterial disease [12]. As a WHO Collaborating Center for New Vaccines Surveillance (WHO CC NVs) for new vaccines surveillance in the West African region of the Invasive Bacterial Vaccine Preventable Disease (IB-VPD) network, the Medical Research Council Unit The Gambia at the London School of Hygiene and Tropical Medicine (MRCG at LSHTM), provides laboratory support for pediatric bacterial meningitis (PBM) surveillance in 10 countries and 17 sentinel hospitals across West and Central Africa.

In July 2008, Togo introduced a pentavalent vaccine against diphtheria, whooping cough, tetanus, hepatitis B, and Hib into the Expanded Program on Immunization (EPI), which is administered in 3 doses to infants at 6, 10, and 14 weeks after birth [13]. Coverage rates for the pentavalent vaccine, estimated by the WHO and United Nations Children’s Fund (UNICEF), have been increasing annually, reaching 90% in 2017 [14]. The pivotal role of pneumococcus in contributing to bacterial meningitis cases was highlighted in a study investigating the burden of disease in Northern Togo prior to the PCV introduction [15]. A second study evaluated pneumococcal serotype distribution within Togo from 2007 to 2009 and found that the 10-valent and 13-valent PCVs incorporated up to 53% of the serotypes responsible for PBM cases [16].

Thus, in 2014, introduction of the 13-valent PCV (PCV13) targeting 13 invasive pneumococcal serotypes (1, 3, 4, 5, 6A, 6B, 7F, 9V, 14, 18C, 19A, 19F, and 23F), has prevented pneumococcal meningitis epidemics within the country [16, 17]. PCV13 is also administered to infants in 3 doses at 6, 10, and 14 weeks after birth, and the WHO and UNICEF immunization coverage estimates are also encouraging, reporting 90% coverage in 2017 [13, 14]. Additionally, in 2014, Togo was part of a vaccination campaign with the MenAfriVac, providing coverage for up to 85% of children at risk of invasive bacterial disease (IBD) [2]. In addition, during the recent outbreak of 2016, Gavi provided MenAfriVac vaccine doses for vaccination of more than 400 000 people aged under 2 years to 29 years in Togo [18].

Togo is one of the smallest countries in Africa and has a population of approximately 7.6 million people [19]. The human immunodeficiency virus (HIV) prevalence in adults aged 15–49 years is 2.1% with approximately 110 000 adults and children living with HIV [20]. Togo is divided into 5 regions, the northernmost, bordering with Burkina Faso is the Savanes region, followed by Kara, Centrale, Plateaux, and the southernmost, Maritime region, which meets the North Atlantic Ocean and is home to the capital city Lomé. Current surveillance of bacterial meningitis within Togo show that the heaviest burden of disease occurs in the Savanes, Kara, and Centrale regions [21]. However, the outbreak in early 2017, caused by meningococcus (serogroup W), was localized to the Plateaux region [2]. Meningitis surveillance data are scarce for the Maritime Region; therefore, this surveillance site was established to assess the prevalence of the most common causes of bacterial meningitis in children aged <5 years here. We report the trends of IBD and pathogens detected in samples collected over a 7-year period, from 2010 to 2016, as part of efforts to monitor the impact of the PCV13, MenAfriVac, and Hib vaccines within Togo.

## METHODS

### Surveillance Structure and Design

Routine surveillance of children <5 years of age admitted to the Sylvanus Olympio University Hospital Center (CHU SO) in Lomé with suspected bacterial meningitis, was carried out from 2010 to 2016. During analysis, July 2010 to June 2014 was considered the prevaccine period and July 2014 to June 2016 the postvaccine period for both the PCV13 and MenAfriVac vaccination. As the Hib vaccine was introduced in Togo prior to this surveillance period, pre- and postvaccine introduction comparisons could not be made.

### Case Enrollment

Suspected meningitis cases were defined, following WHO guidelines, by a sudden onset of fever (>38° C axillary or >38.5° C rectal temperatures), in combination with any of the following clinical symptoms: altered consciousness, stiffness in the head/neck, sensitivity to light, and bulging fontanel [22]. Confirmed bacterial meningitis cases were defined by the identification of pneumococcus, *H. influenzae,* or meningococcus in the cerebrospinal fluid (CSF) of a suspected case [22]. A lumbar puncture (LP) was performed, for children admitted to the CHU SO who met the standard case definition, for routine diagnostic tests, and CSF collected.

### CSF Analysis

Within the CHU SO microbiology laboratory CSF samples were processed following the WHO standard operating procedure [22]. Briefly, samples were centrifuged, and the resulting bacterial pellet was used to prepare smears for Gram staining. Latex agglutination was performed using the supernatant, with the Pastorex meningitis kit (Biorad, Watford, UK) for the detection of pneumococcus, *H. influenzae* and meningococcus. When available, a BINAX® NOW kit (Alere Inc., Waltham, MA USA) was used to confirm the presence of pneumococcal antigen. A white blood cell (WBC) count was performed, and protein and glucose levels were measured using the trichloroacetic acid turbidimetric and glucose oxidase methods [23, 24].

A loopful of CSF was streaked directly onto Columbia blood agar and chocolate agar plates enriched with 5% defibrillated sheep blood. Following overnight incubation, plates were examined for characteristic bacterial growth. Suspected pneumococcal isolates were confirmed via an optochin test (5 μg optochin disk; Oxoid, Basingstoke, UK), whereas suspected *H. influenzae* and meningococcal isolates underwent an analytical profile index (API NH; Biomerieux, Basingstroke, UK). Where possible, serotyping and serogrouping for isolates was performed using various latex agglutination techniques, as previously described [25, 26].

### Molecular Analysis

Approximately 1 mL of CSF from positive meningitis cases were sent to the MRCG at LSHTM for confirmatory testing. Species-specific real-time polymerase chain reaction (RT-PCR) assays were conducted with the following gene targets: *lytA* for *S. pneumoniae*, *hpd* for *H. influenzae,* and *sodC* for *N. meningitidis* [3, 27]. The amplification process consisted of an initial denaturation step at 95° C (10 minutes), followed by 45 cycles of 95° C (15 seconds) and 60° C for 1 minute. Samples with a cycle threshold (CT) value of ≤36 were considered positive.

### Serogrouping and Serotyping

For pneumococcal serotyping, CSF of LytA positive samples was added to TE buffer containing 0.08 g/mL of lysozyme (Sigma-L-6876) and 150 U/mL of mutanolysin (Sigma M-9901) and incubated for 1 hour at 37° C. Nucleic acid extraction was carried out following Qiagen DNA Mini-kit manufacturer instructions and purified DNA underwent sequential triplex real-time PCR assay to detect 21 capsular serotypes as described elsewhere [28]. Additionally, samples with CT values ≤32 were classified as nontypeable and underwent conventional multiplex PCR analysis [29]. Meningococcal gene targets included *sacB, synD, synE, synG, xcbB, synF* for serogroups A, B, C, W, X, and Y, respectively. For *H. influenzae,* gene targets included *acsB, bcsB, ccsD, dscE, ecsH,* and *bexD* for serotypes Hia, Hib, Hic, Hid, Hie, and Hif, respectively. CT values of ≤32 were considered positive for both meningococcus and *H. influenzae* [30].

### Antimicrobial Susceptibility Testing and Whole Genome Sequencing

Antibiotic susceptibility tests were performed on pneumococcal isolates via Kirby-Bauer disc diffusion methods, and resistance was confirmed via inhibition zones interpreted according to the Clinical and Laboratory Standard Institute guidelines [31]. The following antibiotics were tested: rifampicin (RD), erythromycin (E), ceftriaxone/cefotaxime (CTX), tetracycline (TE), vancomycin (VA), oxacillin (OX), chloramphenicol (C), trimethoprim/sulfamethoxazol (SXT), meropenem (MEM), and clindamycin (DA).

Genomic DNA was extracted using a modified Qiagen kit protocol and sent to the Wellcome Trust Sanger Institute for sequencing on an Illumina HiSeq using in-house pipelines [32]. Sequencing reads from isolates were mapped onto the *S. pneumoniae* ATCC_700669 serotype 23F reference genome using SMALT and pseudo genomes were placed in a multiple sequence alignment using custom scripts. Single nucleotide polymorphisms (SNPs) were called from the pseudo alignment using SNP sites, and a maximum likelihood phylogeny was constructed with a general time reversible model using RAxML [33]. The phylogenetic tree was visualized and annotated using the interactive tree of life (iTol) software [34].

### Statistical Analysis

Data were collected at the CHU SO in Lomé using standardized WHO Regional Office for Africa (Afro) PBM network case report forms. Information recorded included patient demographics, clinical symptoms, vaccination history, and laboratory information (CSF microscopy, bacteriological tests, genotyping). Data were then added to a WHO Epi Info-based customized new-vaccine surveillance data module. Data cleaning and analysis were first performed at the CHU SO and then sent to national and regional WHO data managers. For presentation here, data were analyzed using GraphPad Prism 8.1.1. Percentages, proportion, means, and standard deviations were calculated as appropriate and presented as prose, tables, and figures.

### Ethical Approval

Ethical approval was not a requirement in Togo for routine meningitis surveillance including drug susceptibility testing of collected. However, informed consent was sought from caregivers of the surveillance participants. Additionally, the surveillance received overarching ethical approval (SCC1188) by the joint MRC/The Gambia Government ethics board that allowed the analysis of collected West African isolates at MRCG.

## RESULTS

### Demographic and Clinical Characteristics

A summary of the demographic and clinical characteristics of the children enrolled in this surveillance is shown in Table 1. A total of 3644 children <5 years old were admitted to the CHU SO with suspected meningitis from 2010 to 2016. From these patients, 3546 (97.3%) CSF samples were collected and tested at the MRCG at LSHTM. The median age of the patients was 22 months (interquartile range: 5–36 months). The greatest number of suspected cases were seen in children aged 24–59 months (46.9%; 1708/3644). Overall, 56.9% (2075/3644) of patients were male, and the mortality rate for those with suspected meningitis was 6.9% (252/3644).

**Table 1.** Summary of the Clinical and Demographic Characteristics of Suspected Meningitis Cases in Children <5 Years, Maritime Region, Togo, 2010–2016 (N = 3644)

Characteristic	Category	Total	
		n	%
Age	0–11 m	1421	39.0
	12–23 m	512	14.1
	24–59 m	1708	47.0
	Unknown	3	0.1
Sex	Female	1567	43.0
	Male	2075	57.0
	Unknown	2	0.1
Antibiotic before admission	Yes	593	16.3
	No	897	24.6
	Unknown	2154	59.1
Outcome diagnosis	Meningitis	423	11.6
	Pneumonia	170	4.7
	Septicemia	21	0.6
	Other/multiple	1512	41.5
	Unknown	1518	41.7
Outcome	Discharged alive	2060	56.5
	Died	252	6.9
	Unknown	1332	36.6
Case type^a^	Suspected	3644	100.0

^a^Suspected cases include cases that were defined as suspected, probable, and confirmed as per World Health Organization case definition guidelines [22].

The clinical characteristics of patients enrolled in sentinel surveillance are detailed in Table 2. In summary, 41 (1.4%: 41/2912) children who presented with clear CSF samples had confirmed bacterial meningitis. However, a higher proportion of patients (57/659:8.6%) with turbid, xanthochromic, and blood-stained CSF samples were infected with IBD pathogens. The percentage of patients with PBM decreased with WBC count; 45% (36/80) patients with a high WBC count (>100 cells/mm^3^) had PBM compared to 10.2% (21/206) and 1.3% (41/3206) of patients with WBC counts of >10 to 100 cells/mm^3^ and ≤10 cells/mm^3^, respectively. More patients with PBM had high levels of protein (>100 mg/dL) in their CSF (9.5%:21/241) compared to those with low protein levels ≤100 mg/dL (1.5%: 28/1890). However, a higher proportion of children with CSF glucose levels of ≤40 g/dL had PBM (11.2%: 21/187) compared to (1.5 %: 30/1944) patients with higher lower glucose levels (>40 g/dL).

**Table 2.** Summary of Clinical Characteristics of Patients in Relation to Causative Pathogen

		Recruited	Tested	Pneumococcus	Meningococcus	*Haemophilus influenzae*
Characteristic		N	n (%)	n (%)	n (%)	n (%)
CSF appearance	Clear	2912	2888 (99.2)	18 (0.6)	9 (0.3)	14 (0.5)
	Turbid	192	191 (99.5)	42 (21.9)	0	8 (4.1)
	Xanthrochromic	108	108 (100.0)	2 (1.9)	0	0
	Blood Stained	359	357 (99.4)	4 (1.1)	0	1 (0.3)
	Unknown	73	2 (2.7)	0	0	0
White blood cell count (cells/mm^3^)	≤10	3206	3203 (99.9)	22 (0.7)	4 (0.1)	15 (0.5)
	>10 to 100	206	206 (100.0)	14 (6.8)	5 (2.4)	2 (1.0)
	>100	80	80 (100.0)	30 (37.5)	0	6 (7.5)
	Unknown	152	57 (37.5)	0	0	0
Protein (mg/dL)	≤100	1890	1878 (99.4)	9 (0.5)	7 (0.4)	12 (0.6)
	>100	241	241 (100.0)	18 (7.5)	0	5 (2.0)
	Unknown	1513	1427 (94.3)	39 (2.7)	2 (0.1)	6 (0.4)
Glucose (g/dL)	≤40	187	186 (99.5)	16 (8.6)	1 (0.5)	4 (2.1)
	≥40	1944	1934 (99.5)	11 (0.6)	6 (0.3)	13 (0.67)
	Unknown	1513	1426 (94.2)	39 (2.7)	2 (0.1)	6 (0.4)
Total number of suspected cases recruited^a^		3644	3546 (97.3)	66 (1.9)	9 (0.25)	23 (0.65)

Abbreviation: CSF, cerebrospinal fluid.

^a^Suspected cases include cases that were defined as suspected, probable, and confirmed as per World Health Organization case definition guidelines [22].

The case-fatality rate of children with confirmed bacterial meningitis admitted to the CHU SO varied depending on the causative pathogen. For instance, pneumococcus was responsible for 66 cases of PBM identified during surveillance, and from these there was a mortality rate of 25.8% (17/66). However, both meningococcus and *H. influenzae* had lower case fatality rates of 11.1% (1/9) and 13.0% (3/23), respectively. The overall mortality rate for confirmed bacterial meningitis cases was 21.4% (21/98).

### Distribution of Bacterial Pathogens

**Figure 1.**
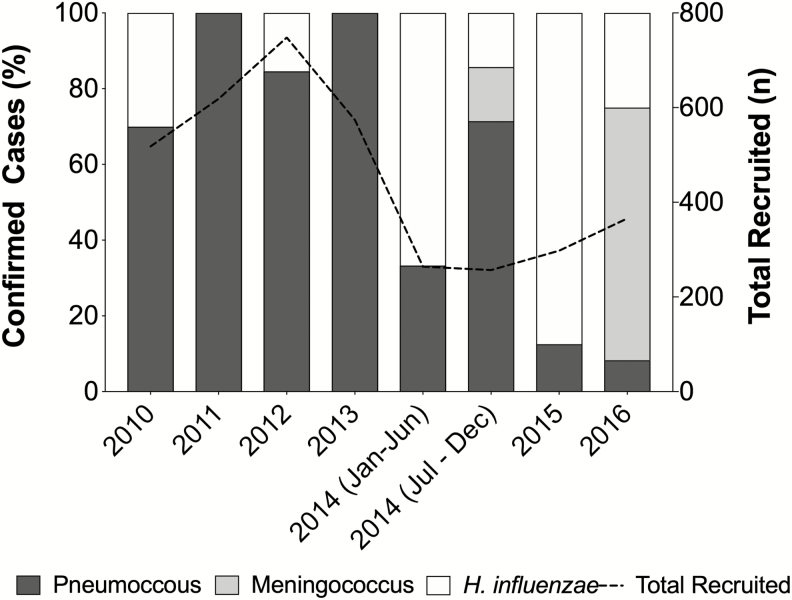
Distribution of suspected pediatric bacterial meningitis cases within Togolese children <5 years from 2010 to 2016. Proportion of pediatric bacterial meningitis cases caused by *Streptococcus pneumoniae* (pneumococcus), *Haemophilus influenzae,* and *Neisseria meningitidis* (meningococcus) seen at the Sylvanus Olympio University Hospital Center in Lomé, Togo. The total number of suspected meningitis cases (N** = **3644) recruited each year during surveillance is indicated by a dashed black line.

The annual number of enrolled suspected meningitis cases varied each year, ranging from 298 to 748 between 2010 and 2016 (Figure 1). The average number of suspected cases annually was 520.6. The prevalence of PBM in the prevaccine period was 2.6% (71/2723) and 2.9% (27/921) in the postvaccine period.

Pneumococcus was the major causative agent, accounting for 67.3% (66/98) of confirmed PBM cases (Figure 1). In the prevaccine period, there were a total of 59 pneumococcal meningitis cases identified with an average of 11.8 cases per year. In comparison, from July 2014 onward (post-PCV13 period) there were 7 cases of pneumococcal meningitis with an average of 2.3 cases per year. This amounts to an 88.1% (52/59) decrease in the number of pneumococcal meningitis cases seen in the postvaccine period compared to the prevaccine period. The overall prevalence of pneumococcal meningitis in the prevaccine period was 2.2% (59/2723) and declined to 0.8% (7/921) post-PCV13 introduction.

Serotype analysis was attempted on 35 pneumococcal isolates received at the MRCG at LSHTM. In the pre-PCV13 period, 2010 to June 2014, serotype analysis was performed on 30 samples, one was nontypeable by PCR, 27 were caused by serotypes targeted by PCV13, and 2 were serotype 15B, which is a non-PCV13 strain. Postvaccine introduction, 5 samples were serotyped, 2 were nontypeable by PCR, and the remaining 3 were caused by PCV13 serotypes 6A and 19F. Prior to PCV13 introduction, pneumococcal serotype 1 was most commonly observed, responsible for 9 PBM cases, followed by 23F, which caused 4 cases. Following vaccine introduction, there were 3 cases of PBM caused by serotypes targeted by the vaccine, compared to 27 cases in the prevaccine period.

During surveillance, there were 23 (23.5%) cases of PBM caused by *H. influenzae* species, and serotyping was performed for 20 isolates at the MRCG at LSHTM. Hib was responsible for 9 (39.1%) cases: 5 cases in 2010, 3 in 2014, and 1 in 2015. After 2011, there was a change in *H. influenzae* serotype distribution observed, with Hic responsible for all cases observed in 2012 (2/2) and Hie accounting for 71.4% (5/7) of cases in 2015. Meningococcus was the least prevalent IBD pathogen observed throughout the surveillance in Togo, accounting for 9 (9.2%) PBM cases. Of these, 8 were observed in 2016 and 1 in 2014; only 1 meningococcal isolate was serogrouped, with a nongroupable result.

### Antimicrobial Susceptibility and Whole Genome Phylogeny of *S. pneumoniae*

**Figure 2.**
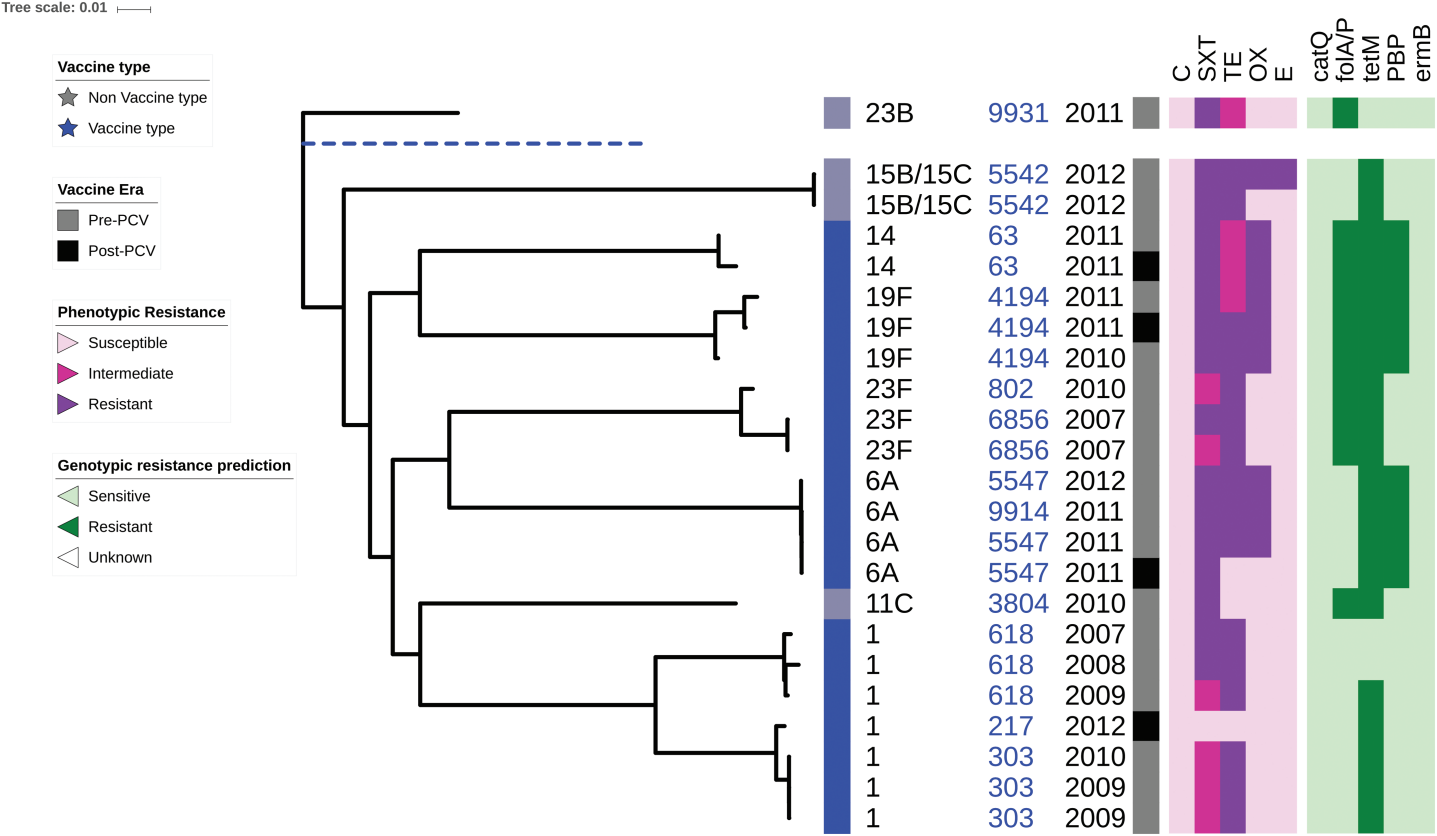
Maximum likelihood whole genome phylogenetic tree of pneumococcal isolates recovered from cerebrospinal fluid pediatric patients in Togo. The tree shows the antibiotic resistance profiles to chloramphenicol (C), trimethoprim/sulfamethoxazol (SXT), tetracycline (TE), oxacillin (OX), and erythromycin (E), of 23 pneumococcal isolates obtained from the cerebrospinal fluid of pediatric bacterial meningitis patients at the Sylvanus Olympio University Hospital Center in Lomé, Togo. Listed in the tree annotations from left to right are the isolates serotype, multilocus sequence type (blue), and year of collection. The key indicates the vaccine type, vaccine era, phenotypic antibiotic resistance, and genotypic antibiotic resistance prediction. A blue dashed line indicates the reference genome (*Streptococcus pneumoniae* ATCC_700669 serotype). Abbreviation: PCV, pneumococcal conjugate vaccine.

A total of 23 pneumococcal isolates obtained from patient CSF samples were whole genome sequenced, including both vaccine and nonvaccine serotypes. The phylogeny reveals a close relationship between strains belonging to the same serotype, particularly among vaccine serotypes such as serotypes 14, 19F, 23F, and 6A (Figure 2). The most common sequence types (STs) in the data set were ST303, ST618 of serotype 1, plus ST5547 and ST4194 of serotypes 6A and 19F, respectively. The nonvaccine serotypes including 15B/15C, 23B, and 11C were isolated mainly in the pre-PCV13 era (Figure 2).

There were 16 isolates (69.6%: 16/23) with antibiotic resistance and 6 (26.1%: 6/23) with intermediate resistance to trimethoprim/sulfamethoxazole. For tetracycline, there were 18 (78.2%: 18/23) resistant isolates and 4 (17.4%: 4/23) intermediate resistant isolates. There was fewer isolates with resistance to oxacillin and erythromycin, 9 (39.1%: 9/23), and 1 (4.4%: 1/23), respectively (Figure 2).

## DISCUSSION

We report data from routine PBM sentinel surveillance carried out at the CHU SO in Lomé, Togo, from 2010 to 2016 and provide data on the impact of PCV13, which was introduced to Togo’s EPI in July 2014. We provide evidence of a decline in the number of children hospitalized due to pneumococcal meningitis post-PCV13 introduction compared to the prevaccine period. For instance, from July 2010 to June 2014 there were 59 cases (2.2%: 59/2723) of pneumococcal meningitis observed in children <5 years at the CHU SO. However, in the post-PCV13 period from July 2014 until the end of surveillance in June 2016, there were 7 cases (0.8%: 7/921) of pediatric pneumococcal meningitis, which is an 88.1% (52/59) reduction in cases.

Pneumococcus was the predominant etiologic agent found in confirmed meningitis cases within pediatric patients from the Maritime region of Togo. The total number of cases caused by PCV13 serotypes decreased over the course of surveillance, and 3 nontypeable (by sequential triplex real-time PCR) serotype cases were detected, 1 in 2012 and 2 in July 2014. Widespread use of PCVs can markedly reduce carriage of vaccine serotypes among both vaccinated and unvaccinated individuals due to decreased transmission from the vaccinated population [35, 36]. Recently, a population based study in The Gambia showed that the incidence of PCV13 serotype invasive pneumococcal disease has reduced significantly, from >200 cases/10^5^ to <50 cases/10^5^ in children aged 2–23 months [7, 37]. Here we report similar findings; a reduction in the number of pneumococcal meningitis cases observed in children <59 months in Lomé, Togo, after the introduction of PCV13.

However, throughout our surveillance, 96.8% (3528/3644) of patients PCV13 vaccination status was either not recorded or unknown. Thus, none of the patients with confirmed pneumococcal meningitis reported receiving a full 3-dose course of the PCV13 vaccine. In fact, the PCV13 vaccination status was not recorded for 96.9% (95/98) of the patients with confirmed bacterial meningitis. Of the pneumococcal isolates we serotyped, 45.5% (30/66) were strains that are included in PCV13 and thus may have been prevented by immunization. Our findings support the need to continue widespread vaccination of Togolese children against pneumococcus and continue surveillance to monitor the burden of vaccine-preventable bacterial meningitis and any potential changes in serotype distribution over time. Encouragingly, the 2017 WHO and UNICEF national immunization coverage estimates suggest that coverage rates for PCV13 are 90% in Togo [14]. Thus, we anticipate that as vaccine coverage increases and herd effect extends to older children and adults, there will be even greater declines in pneumococcal meningitis cases in the Maritime region of Togo.

Our data show that Hib remains responsible for a number of meningitis cases in Togo, despite the use of Hib-containing vaccine and high coverage rates [14]. During surveillance, 67.3% (2453/3644) of patients with suspected meningitis reported receiving the Hib vaccine, 103 of these (4.2%: 103/2453) reported receiving a full 3-dose course of the vaccine. Of the 98 confirmed meningitis cases, 69.4% (68/98) reported receiving at least one dose of the Hib vaccine, but 16 of these patients had confirmed *H. influenzae* meningitis. There were 6 cases of Hib meningitis confirmed in patients who reported receiving the Hib vaccine; however, details of the dates these vaccinations were received were unknown, and vaccination history was provided verbally by next of kin. Overall, we found post-2011, non-Hib (by sequential triplex real-time PCR) serotypes were the most abundant within *H. influenzae* meningitis cases. Conversely, in 2010, we observed the greatest number of Hib confirmed meningitis cases over the entire 7-year surveillance period. These findings may be due to the routine administration of the Hib-containing vaccine within Togo since 2008, leading to a gradual decline in the prevalence of Hib and a greater incidence of non-Hib serotypes causing meningitis or could be due to case ascertainment and serotyping techniques improving so that non-Hib serotype cases are more accurately diagnosed. However, the observation of non-Hib cases of paediatric meningitis within Togo, 7 years after Hib vaccine introduction, is significant and warrants further investigation through continued surveillance as the number of Hib cases continues to decline.

Meningococcus was responsible for just 9 cases of PBM over the surveillance period within the Maritime region of Togo. This result contrasts with reports from the northern and central regions of Togo; meningococcus has been responsible for consecutive meningitis outbreaks in the Savanes, Kara, and Centrale regions [21]. Additionally, in January 2017, meningococcus caused an outbreak in the Plateau region, next to the Maritime region where most of the suspected cases originated from [38]. This difference in the prevalence of IBD pathogens across Togo may be due to geographical, socioeconomic, and other predisposing factors that are distributed differently in different regions of the country.

Overall, of 3644 of suspected bacterial meningitis cases reported during surveillance, only 2.7% (98/3644) were confirmed bacterial meningitis. The WHO case definition for suspected meningitis includes common symptoms that are often associated with other infections, such as severe malaria and viral meningitis, but are still recorded as suspected bacterial meningitis [22]. The WHO case definition for confirmed bacterial meningitis is much more specific; hence, the number of confirmed cases here are much lower in comparison to suspected cases [22]. During surveillance, we focused on 3 major causative agents of bacterial meningitis (pneumococcus, meningococcus, and *H. influenzae)*; however, there are a plethora of other meningeal pathogens that may have been responsible for some of the suspected cases we observed. Thus, using techniques to identify greater meningeal pathogens such as TaqMan array cards may be beneficial for future work.

### Limitations

There are some limitations of this surveillance data that may affect the interpretation of the findings. For instance, there was a lack of detailed clinical and demographic data for a large number of patients with suspected meningitis who had a lumbar puncture. For example, the mortality rate of suspected PBM was 6.9% (252/3644), but the outcome at discharge for 36.6% (1332/3644) patients was not recorded, so the number of patients who died may have been higher than reported. Similarly, long-term sequelae were confirmed in <1.0% (21/3644) of patients, but 52.7% (1921:3644) were not followed up after discharge; thus, the number of patients with long-term sequelae may also be higher than reported. Moreover, for 2% (73/3644) patients with suspected meningitis, the appearance of the CSF sample collected was not recorded. Additionally, only 2 of these samples (2.7%: 2/73) were tested for the presence of bacterial pathogens; thus, the cases of confirmed bacterial meningitis may have been higher than reported.

In addition, the number of samples from suspected cases that were sent to the MRCG at LSHTM for molecular analysis was lower during the prevaccination period compared to the postvaccination period. This could have reduced the quality of data available for analysis and could underestimate the number of positive cases reported for the earlier period. However, the samples were not purposefully selected based on any particular criteria of the patients, and there was no systematic bias for children whose clinical information was collected and whose samples were received and tested at the MRCG at LSHTM.

## CONCLUSION

Bacterial meningitis due to infection with pneumococcus, meningococcus, and *H. influenzae* species remains a significant cause of mortality and morbidity in the Maritime region of Togo, despite the successful introduction of conjugate vaccines. Enhanced surveillance to further examine the impact of vaccination and to detect cases of PBM rapidly to avoid outbreaks is recommended for early detection of epidemics in other regions of Togo as well as the Maritime region. Moreover, the MRCG at LSHTM should continue to collaborate with the CHU SO and Togo to enhance the quality of data captured in relation to the 3 most prominent pathogens causing meningitis in children <5 years of age.

## Notes

***Invasive Bacterial Disease (IBD) writing group.*** Brenda Kwambana Adams, Senghore Madikay, Effua Usuf, Archibald Worwui, Uzochukwu Egere, Akram Zaman, Catherine Okoi, Florian Gehre, Leopold Tientcheu, Nuredin Ibrahim Mohammed, Felix Dube, Peter Ndow, Sambou M. Suso, Sheikh Jarju, Dam Khan, Chinelo Ebruke, Rowan Bancroft, Jason M. Mwenda, and Martin Antonio.

***Author contributions.*** M. A. and J. M. M. established the World Health Organization (WHO) Regional Office for Africa (Afro) supported Pediatric Bacterial Meningitis Surveillance Network in West Africa. M. A. supervised the overall network including setting-up the sentinel surveillance system. E. T., M. F., D. A., M. A. G., N. T., S. D. T., A. D., Y. A., and D. E. L clinically investigated and recruited the patients at the sentinel sites, collected demographic data, performed microbiological testing, and shipped CSF and bacterial isolates to WHO Collaborating Center at the MRCG at LSHTM for confirmatory testing and molecular analysis supervised by B. K. A. and M. A. B. K. A. and M. A. developed the analysis plan, contributed to analysis and interpretation of data along with the IBD writing group. R. E. B., A. K. S., L. T., and B. K. A. drafted the article along with E. T. All authors contributed to the interpretation of the findings and the writing of the final article.

***Acknowledgments.*** The authors acknowledge the surveillance participants, the Ministry of Health, and National Expanded Program for Immunization in Togo for the guidance and leadership in implementing the new vaccines surveillance.

***Financial support.*** Financial support for sentinel site surveillance was provided by the Togo Ministry of Health, GAVI - the Vaccine Alliance, through a grant to the WHO for the African Paediatric Bacterial Meningitis Surveillance Network.

***Supplement sponsorship.*** This supplement was supported with funds from Gavi, the Vaccine Alliance through The World Health Organization and the CDC Foundation, and The Medical Research Council Unit The Gambia at the London School of Hygiene and Tropical Medicine.

***Potential conflicts of interest.*** All authors: No reported conflicts of interest. All authors have submitted the ICMJE Form for Disclosure of Potential Conflicts of Interest. Conflicts that the editors consider relevant to the content of the manuscript have been disclosed.
